# Pain-Related Emergency Department Visits and Hospitalizations Following Hydrocodone Rescheduling in Metastatic Lung Cancer

**DOI:** 10.3390/curroncol33080495

**Published:** 2026-08-21

**Authors:** Chan Shen, Mohammad Ikram, Shouhao Zhou, Roger Klein, Douglas Leslie, James Douglas Thornton

**Affiliations:** 1 Department of Surgery, College of Medicine, The Pennsylvania State University, Hershey, PA 17033, USA; mikram2@pennstatehealth.psu.edu; 2 Department of Public Health Sciences, College of Medicine, The Pennsylvania State University, Hershey, PA 17033, USA; szhou1@pennstatehealth.psu.edu (S.Z.); dleslie@pennstatehealth.psu.edu (D.L.); 3 Penn State Cancer Institute, Hershey, PA 17033, USA; 4 Department of Economics, Rutgers University, New Brunswick, NJ 08901, USA; rklein@economics.rutgers.edu; 5 Department of Pharmaceutical Health Outcomes and Policy, University of Houston College of Pharmacy, Houston, TX 77204, USA; jdthornt@central.uh.edu; 6 Prescription Drug Misuse Education and Research (PREMIER) Center, University of Houston College of Pharmacy, Houston, TX 77204, USA

**Keywords:** hydrocodone, lung neoplasms, emergency service, hospital, hospitalization, Medicare, analgesics, opioid

## Abstract

**Background:** Emergency department (ED) use for cancer-related pain is common, particularly among patients with metastatic disease who frequently require opioid analgesia. In October 2014, the U.S. Drug Enforcement Administration rescheduled hydrocodone combination products from Schedule III to Schedule II, introducing stricter prescribing and dispensing requirements. We evaluated whether hydrocodone rescheduling was associated with pain-related ED visits and hospitalizations among older adults with metastatic lung cancer. **Methods:** We conducted a retrospective SEER–Medicare cohort study of beneficiaries aged 66 years or older diagnosed with metastatic lung cancer. Diagnoses from January 2011 through September 2014 were classified as pre-policy, October 2014 was excluded as a transition month, and November 2014 through December 2018 constituted the post-policy period. Monthly 365-day cumulative incidences were estimated using the Aalen–Johansen estimator with death treated as a competing event. Segmented interrupted time-series models estimated immediate level and slope changes. Adjusted cause-specific Cox models evaluated time to first event. **Results:** The cohort included 52,371 beneficiaries. For narrowly defined neoplasm-related pain ED visits, the policy was associated with an immediate increase of 0.834 percentage points (95% CI, 0.330–1.338) and a post-policy slope increase of 0.030 percentage points per month (95% CI, 0.011–0.049). Pain-related hospitalizations increased immediately by 0.946 percentage points (95% CI, 0.336–1.556), with a slope increase of 0.024 percentage points per month (95% CI, 0.005–0.042). At 12 months, fitted cumulative incidences exceeded no-policy projections by 1.193 percentage points for ED visits and 1.228 percentage points for hospitalizations. Adjusted cause-specific hazard ratios were 1.14 for pain-related ED visits (95% CI, 1.00–1.30; *p* = 0.054) and 1.14 for hospitalizations (95% CI, 1.00–1.29; *p* = 0.049). **Conclusions:** Hydrocodone rescheduling was temporally associated with modest increases in acute-care encounters explicitly coded for neoplasm-related pain. The findings underscore the importance of preserving timely analgesic access for patients with advanced cancer.

## Simple Summary

In October 2014, hydrocodone combination products were moved to a more restrictive federal drug schedule, which increased prescribing and dispensing requirements. We examined whether this policy change was associated with pain-related emergency department visits and hospitalizations among older adults with metastatic lung cancer. Using national cancer registry and Medicare claims data from 2011 to 2018, we found modest immediate and continuing increases in acute-care encounters specifically coded for cancer-related pain after the policy change. Similar increases were not observed when broader pain definitions were used. Because this was an observational study, the findings do not prove that hydrocodone rescheduling caused the changes. However, they suggest that opioid-safety policies may have unintended consequences for patients with advanced cancer. Future policies should balance efforts to reduce opioid-related harm with the need to preserve timely access to effective cancer-pain treatment.

## 1. Introduction

Emergency department (ED) use among adults with cancer in the United States is both substantial and increasing. Cancer accounts for an estimated 3.9 million ED visits annually, and compared with other ED users, individuals with cancer are typically older and more likely to require hospitalization [1]. Long-term trends indicate a steady rise in acute-care use. Between 2006 and 2012, there were 29.5 million cancer-related ED visits nationwide, with high admission rates for infections, cardiopulmonary complications, and pain [2]. More recent analyses from 2012–2019 documented 35.5 million ED encounters among patients with cancer and identified pain as the most common presenting complaint [3]. Importantly, pain-related visits more than doubled during this period, underscoring the increasing burden of uncontrolled symptoms. These encounters frequently escalate to inpatient care; for example, in 2019, 57.2% of oncology-related ED presentations resulted in hospitalization [4], and national data show more than 12,000 admissions for neoplasm-related pain among older adults from 2012 to 2016 alone [5].

Effective pain control is central to high-quality cancer care, particularly for patients with metastatic disease, among whom the prevalence of moderate-to-severe pain approaches 70% to 80% [6,7,8]. The burden is especially pronounced in advanced lung cancer, which is the leading cause of cancer-related mortality. Prior studies have documented that pain is recorded in up to 85% of patients with advanced lung cancer during the terminal phase [9], and approximately 69% of individuals with stage IV non-small cell lung cancer report moderate-to-severe pain [10]. This high burden of pain in advanced lung cancer reflects tumor-related factors (e.g., bone metastases, nerve involvement) and treatment effects, underscoring the critical need for effective analgesia. Opioid analgesics remain the foundation of treatment under World Health Organization (WHO) guidelines [11]. Yet the clinical imperative to manage cancer-related pain is situated within a broader context of rising concern about opioid misuse, diversion, and overdose. Over the past decade, federal and state regulators have implemented multiple policies intended to mitigate inappropriate opioid prescribing.

One of the most consequential federal actions was the U.S. Drug Enforcement Administration’s rescheduling of hydrocodone combination products from Schedule III to Schedule II in October 2014 [12]. This change eliminated automatic refills and telephone-called prescriptions and required a new written or electronic prescription for each dispensed supply. Although designed to enhance public safety, such restrictions may inadvertently hinder timely access to analgesics among patients with advanced cancer, potentially shifting pain management into emergency and inpatient settings [13,14,15]. Supporting this concern, a national study of Medicare decedents with poor-prognosis cancers found that declining opioid fills near the end of life coincided with a roughly 51% increase in pain-related ED visits from 2007 to 2017 [15], a pattern the authors attributed to evolving clinical guidelines, heightened regulatory scrutiny, patient apprehension about opioid misuse, and growing awareness of opioid-related risks.

Despite these observations, the specific impact of hydrocodone rescheduling on acute-care utilization among patients with advanced lung cancer has not been well characterized. The existing literature has documented reductions in opioid prescribing following the policy change, but no study has directly examined whether the rescheduling influenced pain-related ED visits or hospitalizations in this particularly vulnerable population. Evidence in this area is urgently needed to understand whether a policy intended to improve public safety inadvertently compromised symptom management for patients with advanced disease.

Our study addresses this critical gap by using comprehensive, nationwide data to evaluate the association of hydrocodone rescheduling with pain-related ED visits and hospitalizations among individuals with advanced lung cancer.

## 2. Methods

### 2.1. Data Source

We used linked Surveillance, Epidemiology, and End Results (SEER)–Medicare data, which capture health care utilization among Medicare beneficiaries aged ≥65 years [16]. The database combines population-based registry information on demographics, tumor characteristics, diagnosis, and vital status with Medicare Parts A, B, and D claims for longitudinal assessment of care. SEER covers more than one-third of the U.S. population.

### 2.2. Study Cohort

We identified Medicare beneficiaries aged 66 years or older who were diagnosed with distant-stage (metastatic) lung cancer between 1 January 2011 and 31 December 2018. Patients were required to have continuous Medicare Parts A, B, and D enrollment without health maintenance organization or Medicare Advantage enrollment during the 12 months before diagnosis to ascertain comorbidities and prior utilization [17,18]. We excluded patients identified only through autopsy or death-certificate reporting. Complete 12-month postdiagnosis enrollment was not required because it would exclude patients who died or lost fee-for-service coverage. Follow-up extended from diagnosis until the earliest of the first month without Parts A and B enrollment, the first month with Medicare Advantage or health maintenance organization enrollment, death, 12 months, or the end of available data. Diagnoses from January 2011 through September 2014 comprised the pre-policy period. October 2014, when hydrocodone combination products moved from Schedule III to Schedule II, was excluded as a transition month; November 2014 through December 2018 comprised the post-policy period.

### 2.3. Pain-Related ED Visits and Hospitalizations

The primary outcomes were the first pain-related ED visit and the first pain-related inpatient hospitalization occurring within 12 months after lung-cancer diagnosis and before loss of observable medical coverage. The primary narrow definition required ICD-9-CM code 338.3 or ICD-10-CM code G89.3 in any diagnosis position on the same ED encounter or inpatient admission.

We identified ED encounters using outpatient revenue-center codes 0450–0459 or 0981, carrier claims with place-of-service code 23, evaluation and management codes 99281–99285, or an ED charge on an inpatient claim. Claims for the same patient and service date were consolidated into one encounter. A pain diagnosis had to appear on that encounter; an unrelated same-day claim did not qualify. We identified inpatient hospitalizations from Medicare inpatient claims and deduplicated them by beneficiary, admission date, and discharge date. A pain diagnosis had to appear on the same admission. We calculated length of stay from admission and discharge dates and examined total inpatient nights in a supplementary analysis. After deduplication within each category, an ED visit and hospitalization occurring on the same date were retained as distinct events in their respective outcome analyses. Detailed claims-based definitions are provided in Supplementary Table S1.

For sensitivity analyses, we adapted the pain component of the Centers for Medicare & Medicaid Services OP-35 measure to construct a broader oncology-relevant definition [3,19]. It included neoplasm-related pain and abdominal, pelvic, chest, back, neck, joint, limb, head, mucosal, and other pain diagnoses.

Follow-up for each outcome began at cancer diagnosis. Patients were classified as experiencing the event, dying first, or being administratively censored. An acute-care encounter on the recorded death date was classified as the event; death was competing only when it preceded the encounter.

### 2.4. Patient Characteristics

Patient characteristics included age at diagnosis; sex; race and ethnicity (non-Hispanic White, non-Hispanic Black, Hispanic, Asian/Pacific Islander or American Indian/Alaska Native, other or multiracial, or unknown); Medicaid dual eligibility. The Charlson Comorbidity Index was calculated from ICD-9-CM, ICD-10-CM, and CPT/HCPCS codes during the 12-month baseline period [18,20]. Depression was included given its relationship with pain and acute care needs [21,22]. Census-based neighborhood measures included percentages without a high school diploma and below the federal poverty level and area-level income quartile. Lung-cancer histology was also considered, including an indicator for small-cell lung cancer based on ICD-O-3 histology codes 8041–8045.

### 2.5. Descriptive and General Statistical Procedures

We summarized patient characteristics, crude outcome frequencies, and 12-month mortality overall and by policy period using frequencies and percentages. We compared patient characteristics across periods using Pearson chi-square tests and absolute standardized mean differences. Given the large sample, we emphasized standardized mean differences; values of 0.10 or greater were considered potentially meaningful [23]. All tests were two-sided with a significance threshold of 0.05. Analyses used SAS version 9.4 and Python version 3.13.

### 2.6. Interrupted Time-Series Analysis

The primary policy analysis used segmented interrupted time-series (ITS) models of monthly diagnosis cohorts [24,25]. For each monthly cohort, the outcome was the 365-day cumulative incidence estimated using the Aalen–Johansen estimator, with death before the outcome treated as a competing event [26,27]. Models were fitted separately for the narrow primary and broad OP-35 outcomes. Weighted segmented regressions included continuous calendar time, a post-policy indicator, a post-policy slope-change term, and month-of-year fixed effects. Models were weighted by monthly cohort size and used Newey–West heteroskedasticity- and autocorrelation-consistent standard errors with 12 monthly lags [28]. The post-policy indicator estimated the immediate level change, and the post-policy time term estimated the monthly slope change. We also estimated the absolute difference between the fitted post-policy trend and projected no-policy counterfactual at 12 months. Results are reported as percentage-point changes with 95% confidence intervals.

ITS sensitivity analyses added an indicator for the October 2015 transition from ICD-9-CM to ICD-10-CM coding and separately excluded patients diagnosed during 2014 and 2015.

### 2.7. Competing-Risk and Time-to-Event Analyses

To provide descriptive absolute risks complementary to the interrupted time-series analysis, we pooled beneficiaries by policy period and used the Aalen–Johansen estimator to estimate the 365-day cumulative incidence of each outcome, with death before the outcome treated as a competing event. We reported the pre-policy and post-policy cumulative incidences and their unadjusted absolute difference.

Using the follow-up and competing-event definitions above, adjusted cause-specific Cox models estimated associations between the post-policy period and time to first pain-related ED visit or hospitalization [27]. Models included continuous calendar time, month of diagnosis, age, sex, race and ethnicity, Medicaid dual eligibility, depression, Charlson Comorbidity Index, neighborhood income quartile, and lung-cancer histology. We report cause-specific hazard ratios with 95% confidence intervals.

The broad outcome defined above and the 2014–2015 exclusion were also evaluated in the Cox models. An additional sensitivity analysis excluded patients with baseline congestive heart failure, renal disease, or AIDS. Exploratory interaction analyses assessed variation by Medicaid dual eligibility, non-Hispanic Black race, depression, and small-cell lung-cancer histology. We interpreted them cautiously given multiple comparisons.

### 2.8. Supplementary Descriptive Analyses

Using the encounter definitions above, we summarized all, narrow pain-related, and broad pain-related ED visits and inpatient admissions and total inpatient nights during observable postdiagnosis medical follow-up. To account for varying follow-up due to death, loss of fee-for-service coverage, or administrative censoring, utilization was reported per 100 observed person-months (eligible days divided by 30.4375), overall and by policy period. Among patients with both first events, we calculated the proportion whose first ED visit and hospitalization occurred on the same date. These analyses were descriptive and unadjusted.

Opioid dispensing was assessed among beneficiaries with at least one observable day of postdiagnosis Medicare Part D follow-up, from diagnosis until loss of Part D enrollment, death, 12 months, or the end of available data. We identified at least one dispensing of any opioid and of hydrocodone, oxycodone, morphine, fentanyl, tramadol, codeine, or hydromorphone. Among opioid users, we classified hydrocodone only, hydrocodone plus an alternative opioid, or alternative opioids without hydrocodone. Observable Part D follow-up was summarized using medians and interquartile ranges by policy period. These analyses were descriptive; postdiagnosis opioid use was not included as a covariate because it followed policy exposure and could lie on the pathway to acute-care utilization.

### 2.9. Ethical Approval

The Institutional Review Board approved this retrospective analysis and waived informed consent because it used deidentified secondary data. It was approved/exempted by the Institutional Review Board of Penn State College of Medicine (protocol code: 00021341; date of approval: 19 October 2022).

## 3. Results

### 3.1. Study Cohort and Sample Characteristics

The cohort included 52,371 beneficiaries: 24,410 diagnosed pre-policy and 27,961 post-policy; October 2014 diagnoses were excluded. Overall, 53.2% were female, 79.7% non-Hispanic White, and 32.2% aged 80 years or older; 31.6% were dually eligible for Medicaid, 30.8% had baseline depression, and 39.2% had a Charlson Comorbidity Index of 3 or higher. Within 12 months, 75.4% died. Most characteristics were similar by period (absolute standardized mean differences < 0.10). Medicaid dual eligibility showed the largest imbalance, decreasing from 35.7% pre-policy to 28.1% post-policy (standardized mean difference, 0.164). Table 1 presents all characteristics.

### 3.2. Descriptive Acute-Care Outcomes

In the full cohort, 3498 patients had a narrow neoplasm-related pain ED visit (5.3% pre-policy vs. 7.9% post-policy), and 3867 had a narrow pain-related hospitalization (6.0% vs. 8.6%). Broad pain-related ED visits occurred in 55.2% and 56.0%, and broad hospitalizations in 27.2% and 28.4%, respectively. Twelve-month mortality was 77.1% pre-policy and 74.0% post-policy.

### 3.3. Interrupted Time-Series Analyses

For narrow pain-related ED visits, interrupted time-series models estimated an immediate increase of 0.834 percentage points (95% CI, 0.330–1.338; *p* = 0.001) and a post-policy slope increase of 0.030 percentage points per month (95% CI, 0.011–0.049; *p* = 0.002). At 12 months, fitted cumulative incidence exceeded the no-policy projection by 1.193 percentage points (95% CI, 0.626–1.759; *p* < 0.001). For narrow pain-related hospitalizations, the immediate increase was 0.946 percentage points (95% CI, 0.336–1.556; *p* = 0.002), and the post-policy slope increase was 0.024 percentage points per month (95% CI, 0.005–0.042; *p* = 0.012). The 12-month difference from the no-policy counterfactual was 1.228 percentage points (95% CI, 0.682–1.775; *p* < 0.001).

Broad OP-35 outcomes showed no significant level or slope changes. For ED visits, the level change was −0.679 percentage points (95% CI, −2.466 to 1.108) and slope change −0.002 percentage points per month (95% CI, −0.063 to 0.060). For hospitalizations, the corresponding estimates were 0.618 percentage points (95% CI, −0.459 to 1.695) and −0.024 percentage points per month (95% CI, −0.070 to 0.022). Twelve-month counterfactual differences were also nonsignificant.

**Figure 1:**
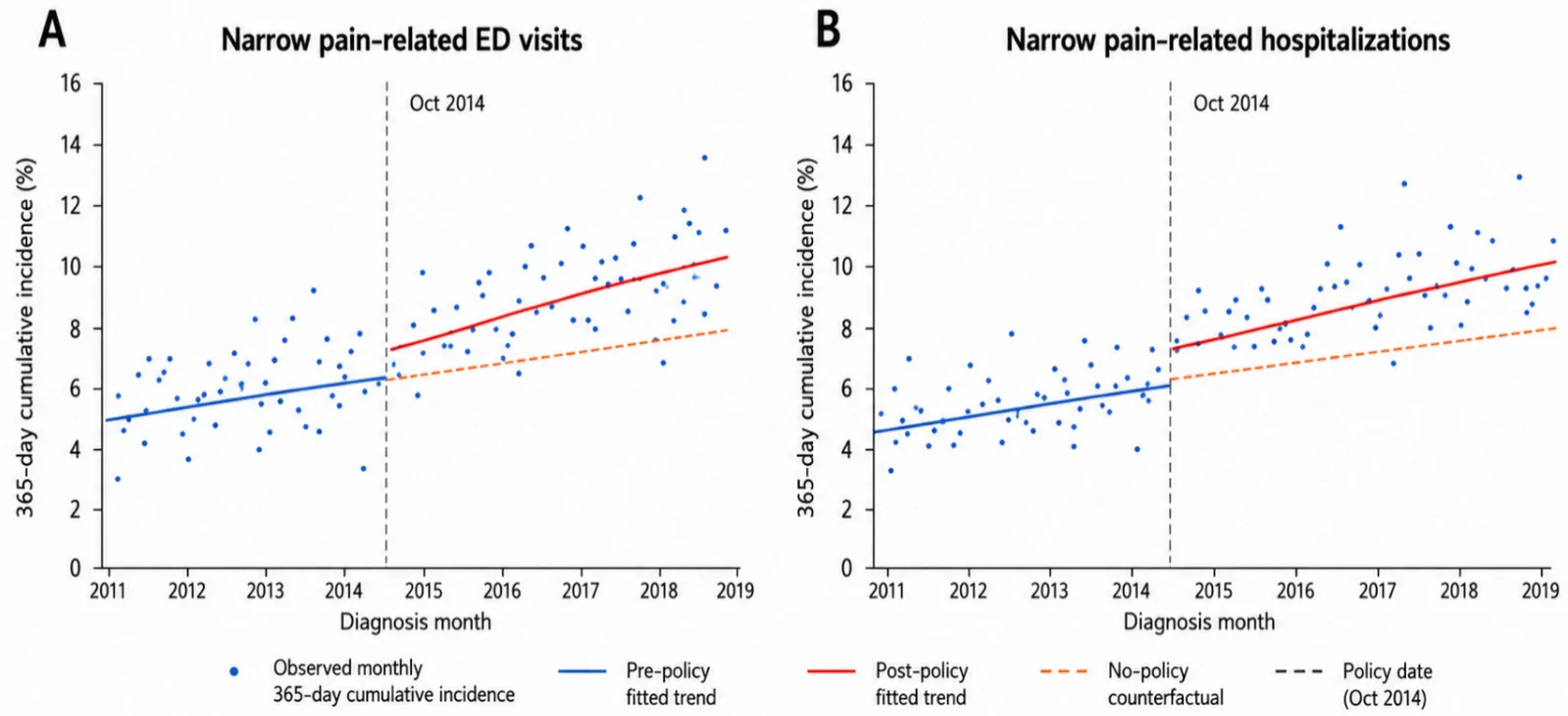
Interrupted time-series plots for narrow pain-related acute-care outcomes. Panel (**A**) shows the monthly observed 365-day cumulative incidence of narrow pain-related emergency department (ED) visits by diagnosis month. Panel (**B**) shows the monthly observed 365-day cumulative incidence of narrow pain-related hospitalizations by diagnosis month. Solid blue lines indicate pre-policy fitted trends; solid red lines indicate post-policy fitted trends from segmented interrupted time-series models; dashed orange lines indicate the no-policy counterfactual projected from the pre-policy trend. The vertical dashed line marks the October 2014 hydrocodone rescheduling policy date. ED, emergency department.

With adjustment for the October 2015 ICD-10-CM transition, narrow-outcome level-change estimates were similar, while slope-change estimates were modestly attenuated and less precise (Supplementary Table S2). Excluding 2014–2015 diagnoses yielded immediate level increases of 1.482 percentage points for narrow ED visits and 1.538 percentage points for narrow hospitalizations (both *p* < 0.001), but nonsignificant slope changes. This specification also yielded significant immediate increases for both broad outcomes and a significant broad ED slope increase. Because the exclusion created a 24-month gap spanning the policy transition, we interpreted these results cautiously (Table 2, Figure 1, and Supplementary Table S2).

### 3.4. Competing-Risk and Time-to-Event Analyses

Accounting for death as a competing event, the unadjusted 365-day cumulative incidence of narrow pain-related ED visits was 5.30% pre-policy and 7.98% post-policy (absolute difference, 2.68 percentage points); corresponding estimates for narrow hospitalizations were 6.06% and 8.65% (absolute difference, 2.58 percentage points). For the broad outcomes, the absolute differences were 0.88 percentage points for ED visits and 1.23 percentage points for hospitalizations (Table 3 and Supplementary Table S3).

Adjusted cause-specific Cox models estimated a 14% higher post-policy hazard of a first narrow pain-related ED visit (HR, 1.14; 95% CI, 1.00–1.30; *p* = 0.054) and hospitalization (HR, 1.14; 95% CI, 1.00–1.29; *p* = 0.049); the ED estimate did not meet the conventional significance threshold. Broad outcomes were not significantly associated with the post-policy period (ED visits: HR, 0.98; 95% CI, 0.94–1.03; *p* = 0.405; hospitalizations: HR, 1.03; 95% CI, 0.97–1.10; *p* = 0.326) (Table 3).

### 3.5. Sensitivity and Subgroup Analyses

After excluding 2014–2015 diagnoses, post-policy HRs among 38,574 patients were 1.34 for narrow ED visits (95% CI, 1.05–1.72; *p* = 0.019) and 1.30 for narrow hospitalizations (95% CI, 1.03–1.64; *p* = 0.029). After excluding baseline congestive heart failure, renal disease, or AIDS among 36,145 patients, estimates were attenuated (ED visits: HR, 1.11; 95% CI, 0.95–1.29; *p* = 0.193; hospitalizations: HR, 1.06; 95% CI, 0.92–1.23; *p* = 0.397) (Table 4).

No statistically significant interaction was observed for Medicaid dual eligibility, non-Hispanic Black race, depression, or small-cell histology; the smallest P value was for depression in the narrow ED model (P for interaction = 0.067). Given multiple unadjusted comparisons, these findings were interpreted cautiously (Supplementary Table S4).

### 3.6. Supplementary Utilization and Opioid-Dispensing Analyses

Per 100 observed person-months, all ED visits increased from 40.39 pre-policy to 43.14 post-policy, narrow pain-related ED visits from 1.04 to 1.65, and broad pain-related ED visits from 16.85 to 17.08. Among patients with both qualifying events, the first ED visit and hospitalization occurred on the same date for 58.6% with any events, 90.0% with narrow events, and 51.5% with broad events. Per 100 person-months, all inpatient admissions decreased from 32.41 to 31.25, narrow pain-related admissions increased from 1.21 to 1.75, and broad pain-related admissions increased from 6.27 to 6.54. Inpatient nights decreased from 264.33 to 247.64. These descriptive analyses were unadjusted (Supplementary Table S5).

Among 52,319 beneficiaries with observable postdiagnosis Part D follow-up, any opioid dispensing was observed in 62.9% pre-policy and 59.5% post-policy. Hydrocodone dispensing decreased from 39.4% to 29.5%, while tramadol increased from 11.7% to 15.5%. Among opioid users, alternative opioid use without hydrocodone increased from 37.5% to 50.4%, and hydrocodone-only use decreased from 26.9% to 20.0% (Supplementary Table S6).

## 4. Discussion

In this national SEER–Medicare cohort of 52,371 older adults with metastatic lung cancer, the primary interrupted time-series analyses identified immediate and progressive increases in narrowly defined neoplasm-related pain acute-care encounters during the period following hydrocodone rescheduling. Patient-level competing-risk analyses were directionally consistent with a modest post-policy elevation in the cause-specific hazard of a first narrowly defined event, although the ED estimate narrowly missed the conventional threshold for statistical significance. In contrast, the primary interrupted time-series models did not identify significant level or slope changes for the broader CMS OP-35 pain outcomes. Collectively, these results suggest a specific change in encounters explicitly coded for neoplasm-related pain rather than a generalized increase in all pain-associated or total acute-care utilization.

The findings add to evidence that access to opioid analgesia for patients with advanced cancer became more constrained during the broader opioid-policy era [15]. Enzinger and colleagues found declines in opioid receipt and dose among Medicare beneficiaries dying of poor-prognosis cancers between 2007 and 2017, while pain-related ED visits increased from 13.2% to 19.9% [15]. Although that study did not isolate hydrocodone rescheduling, our findings extend this literature by identifying a change in narrowly coded cancer-pain ED visits and hospitalizations around the October 2014 policy transition.

Several pathways could plausibly explain this temporal pattern. Moving hydrocodone combination products from Schedule III to Schedule II eliminated refills and increased coordination among patients, caregivers, prescribers, and pharmacies [14]. For patients with rapidly progressive cancer, even brief delays in obtaining a new prescription, locating medication, or arranging reassessment may complicate pain management. Studies have reported reductions in hydrocodone prescribing or dispensing after rescheduling [14,29], sometimes accompanied by increased use of alternative opioids [14].

The descriptive prescription findings in the present study provide compatible, although not causal, context. Hydrocodone dispensing was observed in 39.4% of pre-policy beneficiaries and 29.5% of post-policy beneficiaries with observable Part D follow-up, while use of alternative opioids increased. These results cannot establish whether substitution was clinically adequate or whether acute-care encounters resulted from medication-access problems.

Maintaining access to effective analgesia is particularly important in advanced cancer. A systematic review estimated that approximately two-thirds of patients with advanced, metastatic, or terminal cancer experience pain, with more than half reporting moderate-to-severe pain [30]. Current American Society of Clinical Oncology guidance recommends offering opioids to adults with moderate-to-severe pain from cancer or active cancer treatment unless contraindicated [31]. Consequently, opioid-safety policies must distinguish between inappropriate exposure and the clinical need for timely initiation, renewal, and titration of analgesia in patients with serious illness.

The regulatory environment may also affect cancer-pain management through clinician uncertainty and patient stigma. Qualitative research among patients with advanced cancer and their support persons has documented stigmatizing experiences in healthcare and pharmacy settings, anticipated stigma, guilt, fear of addiction, and behaviors intended to minimize or avoid opioid use [32]. Such experiences may discourage communication about worsening pain or contribute to reluctance to use prescribed opioids [32]. These mechanisms were not measured in SEER–Medicare but may interact with formal prescription requirements.

The absolute magnitude of the findings should be interpreted carefully. The 365-day cumulative incidence of narrow pain-related ED visits was 5.30% before and 7.98% after the policy transition, while hospitalization incidence was 6.06% and 8.65%, respectively. After accounting for the pre-policy trend and seasonality, the modeled 12-month differences from the no-policy counterfactual were approximately 1.2 percentage points for each outcome. These are modest changes at the individual level, but they may have clinical and health-system relevance when applied across a large population with high symptom burden and limited survival. Oncology ED visits frequently involve serious symptoms and commonly lead to hospitalization; recent systematic reviews identify pain as a persistent and prominent driver of acute-care use among patients with cancer [33].

The difference between the narrow and broad outcome findings is important. The narrow codes explicitly identify neoplasm-related pain and therefore provide a clinically specific outcome, but they likely miss cancer pain recorded under less specific diagnoses. The broader OP-35-based definition captures more oncology-relevant pain diagnoses but may include encounters not specifically attributable to neoplasm-related pain [34]. The absence of significant changes in the broad-outcome models suggests that the observed association was concentrated in encounters explicitly identified as neoplasm-related pain. Nevertheless, changes in documentation or coding practices cannot be completely excluded [34].

Accounting for death was a methodological strength because approximately three-quarters of the cohort died within 12 months of diagnosis. The patient-level hazard estimates were modest, reinforcing that the findings should not be presented as evidence of a large policy effect. Results from additional specifications were not uniform, supporting a cautious interpretation. Similar immediate level-change estimates in models accounting for the ICD-10-CM transition reduce concern that the findings were attributable to coding conversion.

The study has several additional strengths. It used a large, national, longitudinal data source; modeled preexisting trends and seasonality; and assessed both ED visits and hospitalizations using consistent encounter-level definitions. Analyses included absolute counterfactual differences as well as relative time-to-event measures, allowing the clinical magnitude of the findings to be evaluated without relying on a simple binary pre–post comparison. The use of the full cohort without requiring survival and continuous enrollment for 12 months reduced selection bias from excluding patients with rapidly progressive disease.

Several limitations remain. First, the observational interrupted time-series design lacked an untreated comparison series and cannot establish that hydrocodone rescheduling caused the observed changes [24]. Other contemporaneous developments, including additional opioid policies, evolving palliative-care delivery, and changes in lung-cancer treatment, may have affected both prescribing and acute-care utilization. Second, claims identify recorded diagnoses rather than pain intensity, clinical appropriateness, medication availability, adherence, prescriber intent, pharmacy refusals, or the timeliness of outpatient symptom management. The study therefore could not determine whether an encounter resulted from undertreated pain, disease progression, treatment toxicity, or another condition, or classify it as avoidable or clinically appropriate. Third, the narrow outcome may underestimate cancer-pain encounters, whereas the broad definition may sacrifice specificity. Finally, the results may not generalize beyond older fee-for-service Medicare beneficiaries in SEER regions.

These findings have practical implications for policy implementation. Opioid safeguards need not be relaxed indiscriminately, but they should be designed so that patients with advanced cancer can obtain clinically appropriate analgesia without avoidable delays [31]. Potential strategies include proactive refill planning, rapid electronic prescribing, pharmacy coordination, after-hours symptom triage, timely palliative-care involvement, and clear exceptions or expedited pathways for patients with serious cancer-related pain [35]. Future studies incorporating prescription-event data, pharmacy-level access measures, patient-reported symptoms, and suitable comparison populations are needed to distinguish medication-access mechanisms from coding and broader secular changes and to assess downstream resource use.

## 5. Conclusions

Hydrocodone rescheduling was temporally associated with immediate and progressive increases in acute-care encounters explicitly coded for neoplasm-related pain among older adults with metastatic lung cancer. The changes were modest, were not reproduced for the primary broad pain outcomes, and do not establish causality. Nevertheless, the findings reinforce the importance of balancing opioid-safety objectives with timely, equitable, and clinically appropriate access to cancer-pain treatment.

## Figures and Tables

**Table 1:** Baseline Characteristics of Medicare Beneficiaries with Metastatic Lung Cancer, Overall and by Hydrocodone Rescheduling Period.

Characteristic	Category	Overall	Pre-Policy	Post-Policy	Maximum	*p*-Value
		(N = 52,371)	(n = 24,410)	(n = 27,961)	Absolute SMD	
**Demographic characteristics**						
Age at diagnosis, years	66–69	9895 (18.9)	4646 (19.0)	5249 (18.8)	0.012	0.534
	70–74	13,790 (26.3)	6463 (26.5)	7327 (26.2)		
	75–79	11,819 (22.6)	5446 (22.3)	6373 (22.8)		
	≥80	16,867 (32.2)	7855 (32.2)	9012 (32.2)		
Sex	Male	24,511 (46.8)	11,441 (46.9)	13,070 (46.7)	0.003	0.772
	Female	27,860 (53.2)	12,969 (53.1)	14,891 (53.3)		
Race/ethnicity	Non-Hispanic White	41,759 (79.7)	19,163 (78.5)	22,596 (80.8)	0.079	<0.001
	Non-Hispanic Black	4350 (8.3)	2169 (8.9)	2181 (7.8)		
	Hispanic	3015 (5.8)	1591 (6.5)	1424 (5.1)		
	Asian/Pacific Islander or American Indian/Alaska Native	2541 (4.9)	1227 (5.0)	1314 (4.7)		
	Other or multiracial	459 (0.9)	214 (0.9)	245 (0.9)		
	Unknown	247 (0.5)	46 (0.2)	201 (0.7)		
**Neighborhood socioeconomic characteristics**						
Census-tract adults without a high-school diploma	0–<5%	4099 (7.8)	1841 (7.5)	2258 (8.1)	0.036	<0.001
	5–<10%	6637 (12.7)	2968 (12.2)	3669 (13.1)		
	10–<20%	12,393 (23.7)	5742 (23.5)	6651 (23.8)		
	≥20%	29,161 (55.7)	13,827 (56.6)	15,334 (54.8)		
	Unknown	81 (0.2)	32 (0.1)	49 (0.2)		
Census-tract population below poverty	0–<5%	11,881 (22.7)	5306 (21.7)	6575 (23.5)	0.043	<0.001
	5–<10%	10,847 (20.7)	5025 (20.6)	5822 (20.8)		
	10–<20%	14,786 (28.2)	6985 (28.6)	7801 (27.9)		
	≥20%	14,776 (28.2)	7062 (28.9)	7714 (27.6)		
	Unknown	81 (0.2)	32 (0.1)	49 (0.2)		
Census-tract median-income quartile	First (lowest)	13,065 (24.9)	6162 (25.2)	6903 (24.7)	0.034	0.001
	Second	13,081 (25.0)	6202 (25.4)	6879 (24.6)		
	Third	13,068 (25.0)	6112 (25.0)	6956 (24.9)		
	Fourth (highest)	13,065 (24.9)	5897 (24.2)	7168 (25.6)		
	Unknown	92 (0.2)	37 (0.2)	55 (0.2)		
**Clinical characteristics**						
Medicaid dual eligibility	No	35,822 (68.4)	15,706 (64.3)	20,116 (71.9)	0.164	<0.001
	Yes	16,549 (31.6)	8704 (35.7)	7845 (28.1)		
Depression	No	36,245 (69.2)	17,044 (69.8)	19,201 (68.7)	0.025	0.004
	Yes	16,126 (30.8)	7366 (30.2)	8760 (31.3)		
Charlson Comorbidity Index	0	8812 (16.8)	4012 (16.4)	4800 (17.2)	0.024	<0.001
	1	11,862 (22.6)	5614 (23.0)	6248 (22.3)		
	2	9840 (18.8)	4706 (19.3)	5134 (18.4)		
	≥3	20,523 (39.2)	9416 (38.6)	11,107 (39.7)		
	Unknown	1334 (2.5)	662 (2.7)	672 (2.4)		
Lung-cancer histology	Non-small-cell/other histology	43,930 (83.9)	20,552 (84.2)	23,378 (83.6)	0.016	0.069
	Small-cell lung cancer	8441 (16.1)	3858 (15.8)	4583 (16.4)		

**Notes:** Values are n (%). The SMD (standardized mean difference) column reports the maximum absolute category-specific standardized mean difference for each characteristic.

**Table 2:** Segmented interrupted time-series estimates of changes in the 365-day cumulative incidence of pain-related acute-care outcomes.

Outcome	Interrupted Time-Series Parameter	Estimate (95% CI)	*p*-Value
Narrow pain-related ED visit	Pre-policy slope, pp/month	0.023 (0.011 to 0.035)	<0.001
	Immediate level change, pp	0.834 (0.330 to 1.338)	0.001
	Change in slope after policy, pp/month	0.030 (0.011 to 0.049)	0.002
	Post-policy slope, pp/month	0.053 (0.038 to 0.068)	<0.001
	Difference from no-policy counterfactual at 12 months, pp	1.193 (0.626 to 1.759)	<0.001
Narrow pain-related hospitalization	Pre-policy slope, pp/month	0.022 (0.013 to 0.031)	<0.001
	Immediate level change, pp	0.946 (0.336 to 1.556)	0.002
	Change in slope after policy, pp/month	0.024 (0.005 to 0.042)	0.012
	Post-policy slope, pp/month	0.046 (0.028 to 0.063)	<0.001
	Difference from no-policy counterfactual at 12 months, pp	1.228 (0.682 to 1.775)	<0.001
Broad pain-related ED visit	Pre-policy slope, pp/month	0.034 (−0.020 to 0.088)	0.219
	Immediate level change, pp	−0.679 (−2.466 to 1.108)	0.456
	Change in slope after policy, pp/month	−0.002 (−0.063 to 0.060)	0.960
	Post-policy slope, pp/month	0.032 (0.008 to 0.057)	0.010
	Difference from no-policy counterfactual at 12 months, pp	−0.698 (−3.078 to 1.683)	0.566
Broad pain-related hospitalization	Pre-policy slope, pp/month	0.025 (−0.003 to 0.053)	0.084
	Immediate level change, pp	0.618 (−0.459 to 1.695)	0.261
	Change in slope after policy, pp/month	−0.024 (−0.070 to 0.022)	0.310
	Post-policy slope, pp/month	0.001 (−0.036 to 0.038)	0.949
	Difference from no-policy counterfactual at 12 months, pp	0.333 (−0.877 to 1.543)	0.590

**Notes:** CI, confidence interval; ED, emergency department; pp, percentage points.

**Table 3:** Competing-risk cumulative incidence and adjusted time-to-first-event associations for pain-related acute-care outcomes.

Outcome	Events, n	365-Day Cumulative Incidence, %			Adjusted Cause-Specific HR (95% CI)	*p*-Value
		Pre-Policy	Post-Policy	Absolute Difference, pp		
Narrow pain-related ED visit	3498	5.30	7.98	2.68	1.14 (1.00 to 1.30)	0.054
Narrow pain-related hospitalization	3867	6.06	8.65	2.58	1.14 (1.00 to 1.29)	0.049
Broad pain-related ED visit	29,111	55.34	56.22	0.88	0.98 (0.94 to 1.03)	0.405
Broad pain-related hospitalization	14,567	27.30	28.53	1.23	1.03 (0.97 to 1.10)	0.326

**Notes:** CI, confidence interval; ED, emergency department; HR, hazard ratio; pp, percentage points.

**Table 4:** Key sensitivity analyses of post-policy associations with time to first narrow pain-related acute-care event.

Outcome	Sensitivity Analysis	N	Events, n	Adjusted Cause-Specific HR (95% CI)	*p*-Value
Narrow pain-related ED visit	Primary full cohort	52,371	3498	1.14 (1.00 to 1.30)	0.054
	Excluding diagnoses in 2014–2015	38,574	2615	1.34 (1.05 to 1.72)	0.019
	Excluding baseline CHF, renal disease, or AIDS	36,145	2619	1.11 (0.95 to 1.29)	0.193
Narrow pain-related hospitalization	Primary full cohort	52,371	3867	1.14 (1.00 to 1.29)	0.049
	Excluding diagnoses in 2014–2015	38,574	2882	1.30 (1.03 to 1.64)	0.029
	Excluding baseline CHF, renal disease, or AIDS	36,145	2904	1.06 (0.92 to 1.23)	0.397

**Notes:** AIDS, acquired immunodeficiency syndrome; CHF, congestive heart failure; CI, confidence interval; ED, emergency department; HR, hazard ratio.

## Data Availability

The datasets analyzed in this article are not readily available because the SEER–Medicare linked data are subject to confidentiality protections and a project-specific Data Use Agreement that prohibits redistribution by investigators. Researchers may request access through the National Cancer Institute’s SEER–Medicare application process. Re-quests to access these datasets should be directed to the SEER–Medicare program.

## Supplementary Materials

The following supporting information can be downloaded at: https://www.mdpi.com/article/10.3390/curroncol33080495/s1, Supplementary Table S1. Claims-Based Definitions and Operational Criteria for Emergency Department Encounters, Inpatient Hospitalizations, and Pain-Related Outcomes; Supplementary Table S2. Complete segmented interrupted time-series estimates for the 365-day cumulative incidence of pain-related acute-care outcomes; Supplementary Table S3. Aalen–Johansen 365-day cumulative-incidence estimates for pain-related acute-care outcomes. Supplementary Table S4. Exploratory interaction analyses of post-policy associations with time to first narrow pain-related acute-care event; Supplementary Table S5. Recurrent Acute-Care Utilization and Inpatient Nights per 100 Observed Medical Person-Months in the Full Cohort; Supplementary Table S6. Observed Postdiagnosis Opioid Dispensing During Available Medicare Part D Follow-Up.

## Abbreviations

SEER	Surveillance, Epidemiology, and End Results
MME	Morphine Milligram Equivalents
NSAIDs	Non-steroidal anti-inflammatory drugs
