# Supplementary material for: Pain-Related Emergency Department Visits and Hospitalizations Following Hydrocodone Rescheduling in Metastatic Lung Cancer
Source: Curr Oncol. 2026 Aug 21;33(8):495. doi: 10.3390/curroncol33080495 (PMC13511036; doi:10.3390/curroncol33080495)
Supplement: Supplementary file 1 [file curroncol-33-00495-s001.zip › curroncol-4385035-supplementary.pdf]

Supplementary Table S1. Claims-Based Definitions and Operational Criteria for Emergency Department Encounters, Inpatient Hospitalizations, and Pain-Related Outcomes

Component	Medicare claim source	Codes or operational criteria	Encounter construction and analytic handling
**ED facility services**	Hospital outpatient claims	Revenue-center codes 0450–0459 or 0981.	Qualifying records were used as evidence of an ED encounter. Records for the same beneficiary and service date were consolidated into one ED encounter.
**ED professional services**	Carrier/professional claims	Place-of-service code 23 (hospital emergency department).	Professional and facility records referring to the same beneficiary and service date were combined and were not counted as separate ED encounters.
**ED evaluation and management services**	Outpatient or professional claims	CPT/HCPCS ED evaluation and management codes 99281–99285.	These codes were accepted as additional evidence of an ED encounter and were consolidated with other qualifying ED records occurring on the same service date.
**ED services associated with an inpatient stay**	Inpatient claims	An ED charge recorded on an inpatient claim.	An ED charge documented on an inpatient claim was retained as an ED encounter; the associated admission was analyzed separately as a hospitalization.
**Inpatient hospitalization**	Medicare inpatient claims	A qualifying inpatient claim with recorded admission and discharge dates.	Admissions were deduplicated by beneficiary, admission date, and discharge date. Length of stay and total inpatient nights were calculated from these dates.
**Primary narrow cancer-pain definition**	Diagnosis fields on the qualifying ED encounter or inpatient admission	ICD-9-CM 338.3 or ICD-10-CM G89.3 (neoplasm-related pain).	The code had to appear on the same encounter or admission. Any diagnosis position was used.
**Broad oncology-pain sensitivity definition**	Diagnosis fields on the qualifying ED encounter or inpatient admission	CMS OP-35 oncology-pain diagnosis set, including neoplasm-related, abdominal, pelvic, chest, back, neck, joint, limb, head, mucosal, and other pain conditions.	The code had to appear on the same encounter or admission. Any diagnosis position was used.
**Same-date and deduplication procedures**	Across outpatient, professional, and inpatient files	Beneficiary and service date for ED encounters; beneficiary, admission date, and discharge date for inpatient admissions.	Multiple ED claims on the same date were counted once. A same-day ED encounter and inpatient admission were retained as distinct service events, but each was counted once within its outcome category. Unrelated same-day claims did not qualify an encounter as pain related.

Notes: CMS, Centers for Medicare & Medicaid Services; CPT, Current Procedural Terminology; ED, emergency department; E/M, evaluation and management; HCPCS, Healthcare Common Procedure Coding System; ICD-9-CM, International Classification of Diseases, Ninth Revision, Clinical Modification; ICD-10-CM, International Classification of Diseases, Tenth Revision, Clinical Modification; OP-35, Admissions and Emergency Department Visits for Patients Receiving Outpatient Chemotherapy.

Supplementary Table S2. Complete segmented interrupted time-series estimates for the 365-day cumulative incidence of pain-related acute-care outcomes.

Outcome	Interrupted time-series parameter	Estimate (95% CI)	p-Value
**Primary model (October 2014 excluded) (95 monthly cohorts)**			
Narrow pain-related ED visit	Pre-policy monthly slope	0.023 (0.011 to 0.035)	<0.001
Narrow pain-related ED visit	Immediate level change	0.834 (0.330 to 1.338)	0.001
Narrow pain-related ED visit	Post-policy slope change	0.030 (0.011 to 0.049)	0.002
Narrow pain-related ED visit	Post-policy monthly slope	0.053 (0.038 to 0.068)	<0.001
Narrow pain-related ED visit	Difference from no-policy counterfactual at 12 months	1.193 (0.626 to 1.759)	<0.001
Narrow pain-related hospitalization	Pre-policy monthly slope	0.022 (0.013 to 0.031)	<0.001
Narrow pain-related hospitalization	Immediate level change	0.946 (0.336 to 1.556)	0.002
Narrow pain-related hospitalization	Post-policy slope change	0.024 (0.005 to 0.042)	0.012
Narrow pain-related hospitalization	Post-policy monthly slope	0.046 (0.028 to 0.063)	<0.001
Narrow pain-related hospitalization	Difference from no-policy counterfactual at 12 months	1.228 (0.682 to 1.775)	<0.001
Broad pain-related ED visit	Pre-policy monthly slope	0.034 (-0.020 to 0.088)	0.219
Broad pain-related ED visit	Immediate level change	-0.679 (-2.466 to 1.108)	0.456
Broad pain-related ED visit	Post-policy slope change	-0.002 (-0.063 to 0.060)	0.960
Broad pain-related ED visit	Post-policy monthly slope	0.032 (0.008 to 0.057)	0.010
Broad pain-related ED visit	Difference from no-policy counterfactual at 12 months	-0.698 (-3.078 to 1.683)	0.566
Broad pain-related hospitalization	Pre-policy monthly slope	0.025 (-0.003 to 0.053)	0.084
Broad pain-related hospitalization	Immediate level change	0.618 (-0.459 to 1.695)	0.261
Broad pain-related hospitalization	Post-policy slope change	-0.024 (-0.070 to 0.022)	0.310

Outcome	Interrupted time-series parameter	Estimate (95% CI)	p-Value
Broad pain-related hospitalization	Post-policy monthly slope	0.001 (-0.036 to 0.038)	0.949
Broad pain-related hospitalization	Difference from no-policy counterfactual at 12 months	0.333 (-0.877 to 1.543)	0.590
**Model additionally adjusted for the October 2015 ICD-10-CM transition (95 monthly cohorts)**			
Narrow pain-related ED visit	Pre-policy monthly slope	0.023 (0.010 to 0.035)	<0.001
Narrow pain-related ED visit	Immediate level change	0.758 (0.244 to 1.273)	0.004
Narrow pain-related ED visit	Post-policy slope change	0.023 (-0.001 to 0.047)	0.058
Narrow pain-related ED visit	Post-policy monthly slope	0.046 (0.024 to 0.067)	<0.001
Narrow pain-related ED visit	Difference from no-policy counterfactual at 12 months	1.035 (0.406 to 1.664)	0.001
Narrow pain-related ED visit	ICD-10-CM transition level change	0.322 (-0.192 to 0.836)	0.220
Narrow pain-related hospitalization	Pre-policy monthly slope	0.022 (0.013 to 0.031)	<0.001
Narrow pain-related hospitalization	Immediate level change	0.905 (0.259 to 1.551)	0.006
Narrow pain-related hospitalization	Post-policy slope change	0.020 (-0.004 to 0.044)	0.099
Narrow pain-related hospitalization	Post-policy monthly slope	0.042 (0.018 to 0.065)	<0.001
Narrow pain-related hospitalization	Difference from no-policy counterfactual at 12 months	1.144 (0.493 to 1.795)	<0.001
Narrow pain-related hospitalization	ICD-10-CM transition level change	0.173 (-0.504 to 0.850)	0.617
Broad pain-related ED visit	Pre-policy monthly slope	0.034 (-0.021 to 0.088)	0.222
Broad pain-related ED visit	Immediate level change	-0.712 (-2.454 to 1.029)	0.423
Broad pain-related ED visit	Post-policy slope change	-0.005 (-0.074 to 0.064)	0.897
Broad pain-related ED visit	Post-policy monthly slope	0.029 (-0.010 to 0.069)	0.146
Broad pain-related ED visit	Difference from no-policy counterfactual at 12 months	-0.767 (-3.139 to 1.605)	0.526

Outcome	Interrupted time-series parameter	Estimate (95% CI)	p-Value
Broad pain-related ED visit	ICD-10-CM transition level change	0.141 (-0.775 to 1.057)	0.762
Broad pain-related hospitalization	Pre-policy monthly slope	0.025 (-0.004 to 0.054)	0.087
Broad pain-related hospitalization	Immediate level change	0.613 (-0.361 to 1.587)	0.217
Broad pain-related hospitalization	Post-policy slope change	-0.024 (-0.085 to 0.037)	0.435
Broad pain-related hospitalization	Post-policy monthly slope	0.001 (-0.056 to 0.058)	0.980
Broad pain-related hospitalization	Difference from no-policy counterfactual at 12 months	0.323 (-0.883 to 1.529)	0.600
Broad pain-related hospitalization	ICD-10-CM transition level change	0.021 (-1.204 to 1.246)	0.973
**Model excluding diagnosis years 2014-2015 (72 monthly cohorts)**			
Narrow pain-related ED visit	Pre-policy monthly slope	0.017 (0.006 to 0.029)	0.004
Narrow pain-related ED visit	Immediate level change	1.482 (0.900 to 2.064)	<0.001
Narrow pain-related ED visit	Post-policy slope change	0.021 (-0.001 to 0.044)	0.066
Narrow pain-related ED visit	Post-policy monthly slope	0.038 (0.019 to 0.058)	<0.001
Narrow pain-related ED visit	Difference from no-policy counterfactual at 12 months	1.735 (1.188 to 2.282)	<0.001
Narrow pain-related hospitalization	Pre-policy monthly slope	0.017 (0.005 to 0.029)	0.005
Narrow pain-related hospitalization	Immediate level change	1.538 (0.944 to 2.132)	<0.001
Narrow pain-related hospitalization	Post-policy slope change	0.015 (-0.008 to 0.038)	0.203
Narrow pain-related hospitalization	Post-policy monthly slope	0.032 (0.014 to 0.051)	<0.001
Narrow pain-related hospitalization	Difference from no-policy counterfactual at 12 months	1.718 (1.154 to 2.281)	<0.001
Broad pain-related ED visit	Pre-policy monthly slope	-0.039 (-0.072 to -0.005)	0.024
Broad pain-related ED visit	Immediate level change	1.846 (0.238 to 3.454)	0.024

Outcome	Interrupted time-series parameter	Estimate (95% CI)	p-Value
Broad pain-related ED visit	Post-policy slope change	0.066 (0.007 to 0.125)	0.027
Broad pain-related ED visit	Post-policy monthly slope	0.027 (-0.019 to 0.073)	0.247
Broad pain-related ED visit	Difference from no-policy counterfactual at 12 months	2.639 (1.057 to 4.220)	0.001
Broad pain-related hospitalization	Pre-policy monthly slope	-0.018 (-0.053 to 0.016)	0.297
Broad pain-related hospitalization	Immediate level change	2.399 (0.677 to 4.122)	0.006
Broad pain-related hospitalization	Post-policy slope change	0.008 (-0.058 to 0.073)	0.816
Broad pain-related hospitalization	Post-policy monthly slope	-0.010 (-0.064 to 0.043)	0.702
Broad pain-related hospitalization	Difference from no-policy counterfactual at 12 months	2.492 (0.793 to 4.192)	0.004

Notes: CI, confidence interval; ED, emergency department; ICD, International Classification of Diseases; pp, percentage points.

Supplementary Table S3. Aalen–Johansen 365-day cumulative-incidence estimates for pain-related acute-care outcomes.

365-day cumulative incidence by policy period

Outcome	Pre-policy cumulative incidence, %	Post-policy cumulative incidence, %	Absolute difference, pp
Narrow pain-related ED visit	5.30	7.98	2.68
Narrow pain-related hospitalization	6.06	8.65	2.58
Broad pain-related ED visit	55.34	56.22	0.88
Broad pain-related hospitalization	27.30	28.53	1.23

Notes: ED, emergency department; pp, percentage points.

Supplementary Table S4. Exploratory interaction analyses of post-policy associations with time to first narrow pain-related acute-care event.

Outcome	Effect modifier	Reference subgroup, adjusted cause-specific HR (95% CI)	Comparison subgroup, adjusted cause-specific HR (95% CI)	p for interaction
**Narrow pain-related ED visit**	Medicaid dual eligibility	No dual eligibility: 1.13 (0.98 to 1.30)	Dual eligibility: 1.16 (0.98 to 1.37)	0.787
	Non-Hispanic Black race	Other race/ethnicity: 1.12 (0.98 to 1.29)	Non-Hispanic Black: 1.30 (1.01 to 1.69)	0.239
	Depression	No depression: 1.08 (0.94 to 1.25)	Depression: 1.24 (1.06 to 1.46)	0.067
	Small-cell lung-cancer histology	Other lung-cancer histology: 1.14 (1.00 to 1.31)	Small-cell lung cancer: 1.13 (0.92 to 1.38)	0.878
**Narrow pain-related hospitalization**	Medicaid dual eligibility	No dual eligibility: 1.14 (1.00 to 1.30)	Dual eligibility: 1.13 (0.96 to 1.33)	0.921
	Non-Hispanic Black race	Other race/ethnicity: 1.13 (1.00 to 1.29)	Non-Hispanic Black: 1.15 (0.90 to 1.48)	0.882
	Depression	No depression: 1.11 (0.96 to 1.27)	Depression: 1.19 (1.02 to 1.38)	0.310
	Small-cell lung-cancer histology	Other lung-cancer histology: 1.14 (1.00 to 1.30)	Small-cell lung cancer: 1.11 (0.92 to 1.34)	0.743

Notes: CI, confidence interval; ED, emergency department; HR, hazard ratio.

Supplementary Table S5. Recurrent Acute-Care Utilization and Inpatient Nights per 100 Observed Medical Person-Months in the Full Cohort

Outcome	Overall, count	Overall rate per 100 person-months	Pre-policy, count	Pre-policy rate per 100 person-months	Post-policy, count	Post-policy rate per 100 person-months	Absolute rate difference per 100 person-months
All ED visits	134,498	41.88	59,507	40.39	74,991	43.14	+2.75
Narrow pain-related ED visits	4,406	1.37	1,533	1.04	2,873	1.65	+0.61
Broad pain-related ED visits	54,509	16.97	24,820	16.85	29,689	17.08	+0.23
All inpatient admissions	102,069	31.78	47,745	32.41	54,324	31.25	-1.15
Narrow pain-related inpatient admissions	4,840	1.51	1,790	1.21	3,050	1.75	+0.54
Broad pain-related inpatient admissions	20,611	6.42	9,242	6.27	11,369	6.54	+0.27
Total inpatient nights	819,868	255.30	389,432	264.33	430,436	247.64	-16.69

Same-day occurrence of first ED visit and hospitalization

Definition	Overall, n/N (%)	Pre-policy, n/N (%)	Post-policy, n/N (%)
Any ED visit and any inpatient admission	23,996/40,939 (58.6)	11,261/18,978 (59.3)	12,735/21,961 (58.0)
Narrow pain-related ED visit and hospitalization	2,722/3,026 (90.0)	985/1,080 (91.2)	1,737/1,946 (89.3)
Broad pain-related ED visit and hospitalization	6,830/13,273 (51.5)	3,163/6,013 (52.6)	3,667/7,260 (50.5)

Notes: ED, emergency department.

Supplementary Table S6. Observed Postdiagnosis Opioid Dispensing During Available Medicare Part D Follow-Up

Measure	Overall (N = 52,319)	Pre-policy (n = 24,381)	Post-policy (n = 27,938)	Difference
**Panel A. Observed dispensing among beneficiaries with at least one observable Part D follow-up day**				
Observable Part D follow-up, median (IQR), days	151 (61-365)	150 (61-335)	151 (61-365)	+1 day
Any opioid	31,962 (61.1)	15,345 (62.9)	16,617 (59.5)	-3.5 pp
Hydrocodone	17,840 (34.1)	9,595 (39.4)	8,245 (29.5)	-9.8 pp
Oxycodone	12,959 (24.8)	5,960 (24.4)	6,999 (25.1)	+0.6 pp
Morphine	6,804 (13.0)	3,254 (13.3)	3,550 (12.7)	-0.6 pp
Fentanyl	5,000 (9.6)	2,659 (10.9)	2,341 (8.4)	-2.5 pp
Tramadol	7,204 (13.8)	2,861 (11.7)	4,343 (15.5)	+3.8 pp
Codeine	3,143 (6.0)	1,356 (5.6)	1,787 (6.4)	+0.8 pp
Hydromorphone	2,720 (5.2)	1,356 (5.6)	1,364 (4.9)	-0.7 pp
**Panel B. Opioid medication pattern among beneficiaries with at least one observed opioid dispensing**				
**Measure**	**Overall (N = 31,962)**	**Pre-policy (n = 15,345)**	**Post-policy (n = 16,617)**	**Difference**
Hydrocodone only	7,452 (23.3)	4,122 (26.9)	3,330 (20.0)	-6.8 pp
Hydrocodone plus at least one alternative opioid	10,388 (32.5)	5,473 (35.7)	4,915 (29.6)	-6.1 pp
Alternative opioid(s) without hydrocodone	14,122 (44.2)	5,750 (37.5)	8,372 (50.4)	+12.9 pp

Notes: IQR, interquartile range; pp, percentage points.
